# Serous retinal detachment after panretinal photocoagulation for proliferative diabetic retinopathy: a case report

**DOI:** 10.1186/s13256-017-1424-y

**Published:** 2017-09-19

**Authors:** Patrik Schatz, Ahmed Aldayel, Ibrahim Taskintuna, Ehab Abdelkader, Marco Mura

**Affiliations:** 1 Vitreoretinal Division, King Khaled Eye Specialist Hospital, Al-Oruba Street, PO Box 7191, Riyadh, 11462 Saudi Arabia; 2 Department of Ophthalmology, Clinical Sciences, Scane County University Hospital, University of Lund, Lund, Sweden; 3 Ophthalmology Department, Menoufia University, Shebin El-Kom, Egypt

**Keywords:** Proliferative diabetic retinopathy, Panretinal photocoagulation, Serous retinal detachment, Optical coherence tomography, Wide-field imaging, Case report

## Abstract

**Background:**

Proliferative diabetic retinopathy is a major cause of visual impairment in working-age adults worldwide. Panretinal photocoagulation is a cornerstone in its management; however, it may include a range of side effects and complications, one of these being serous retinal detachment. To the best of our knowledge, this is the first report of the use of intravitreal injection of bevacizumab for serous retinal detachment after panretinal photocoagulation.

**Case presentation:**

A 24-year-old Saudi man with poorly controlled type 1 diabetes presented with bilateral progressive proliferative retinopathy in spite of several sessions of panretinal photocoagulation. After one additional such session, he developed bilateral serous retinal detachment and vision loss, which was managed with a single bilateral intravitreal bevacizumab injection. The serous retinal detachment subsided with partial recovery of vision.

**Conclusions:**

Serous retinal detachment after panretinal photocoagulation for proliferative diabetic retinopathy is a rare complication nowadays. In this case, it seems that excessive photocoagulation exceeded the energy-absorbing capacity of the retinal pigment epithelium, leading to a disruption of the blood–retinal barrier. A single injection of bilateral intravitreal bevacizumab was sufficient to control the serous retinal detachment. This effect may have been due to a reduction of vascular leakage resulting from the mechanism of action of this drug. No complications were noted from the injection. Caution should be exerted when attempting bilateral panretinal photocoagulation.

## Background

Serous retinal detachment (RD) is a rare complication after panretinal photocoagulation (PRP) for proliferative diabetic retinopathy (PDR) in patients with diabetes mellitus (DM). It is believed to be caused by excessive photocoagulation, exceeding the energy-absorbing capacity of the retinal pigment epithelium, leading to a disruption of the blood–retinal barrier. Persistently elevated glucose levels leading to glycation and other covalent modification of macromolecules, leading to increased oncotic pressure, osmotic gradient, and fluid accumulation in the interstitial tissues, may contribute to this complication. Intensified insulin treatment with improved metabolic control may have reduced the incidence of serous macular detachment after PRP. In an old series including 42 eyes, using an argon laser or xenon arc PRP, 12% or five eyes, developed serous RD [1]. Dividing the PRP into two or more sessions at least 2 weeks apart was noted to reduce the risk of complications associated with PRP [1].

Here we describe a rapidly progressive course of PDR in a poorly regulated patient with type 1 DM, who developed bilateral serous RD after one out of several sessions of PRP.

## Case presentation

A 24-year-old Saudi man with type 1 DM and a history of tobacco smoking presented with bilateral severe non-proliferative diabetic retinopathy (DR). His past medical history was unremarkable except for DM. No ophthalmic interventions, such as laser, intravitreal injections, or surgery, had been given prior to presentation. His social and family history: he had two brothers and three sisters. His mother had a history of type 2 DM and one younger sister had type 1 DM, however, none of them were known to have any DR. Environmental history: he was living in a mainland urban area working as a clerk. He was seen on 12 occasions during 18 months. Compliance with follow-up visits and instructions was variable. On presentation, his temperature was 36 °C and his heart rate was 72. His blood pressure (BP) ranged between 110/60 and 150/90 mmHg, with an average BP of 115/80 mmHg over eight measurements during 16 months. Random blood glucose ranged from 9.8 to 19 mmol/l with an average of 14.6 mmol/l (normal range is 5 to 7 mmol/l in our laboratory). His glycated hemoglobin (HbA1C) was 13.6% (>6.5 is diagnostic of diabetes in our laboratory) or 125 mmol/mol (> 47 is diagnostic of diabetes in our laboratory). Urine analyses and detailed neurological examinations are not routinely performed in our hospital (which is an eye specialist hospital). Our patient was on the following medication prior to presentation and throughout follow-up: Mixtard 30 Novolet (biphasic isophane insulin) subcutaneous injections of 48 Units before midday and 38 Units after midday. His unaided visual acuity was 20/200 in his right eye and 20/300 in his left eye. Fluorescein angiography at baseline (performed with a standard, non-wide-field fundus camera) had demonstrated mild ischemic maculopathy in his right eye with no clinically significant macular edema and severe non-proliferative DR (not shown). His left eye could not be imaged at baseline with the standard fluorescein angiography camera due to poorly dilating pupil. Optical coherence tomography demonstrated a mild macular edema in his left eye (not shown). He was offered, but refused, intravitreal injections in his left eye.

**Fig. 1:**
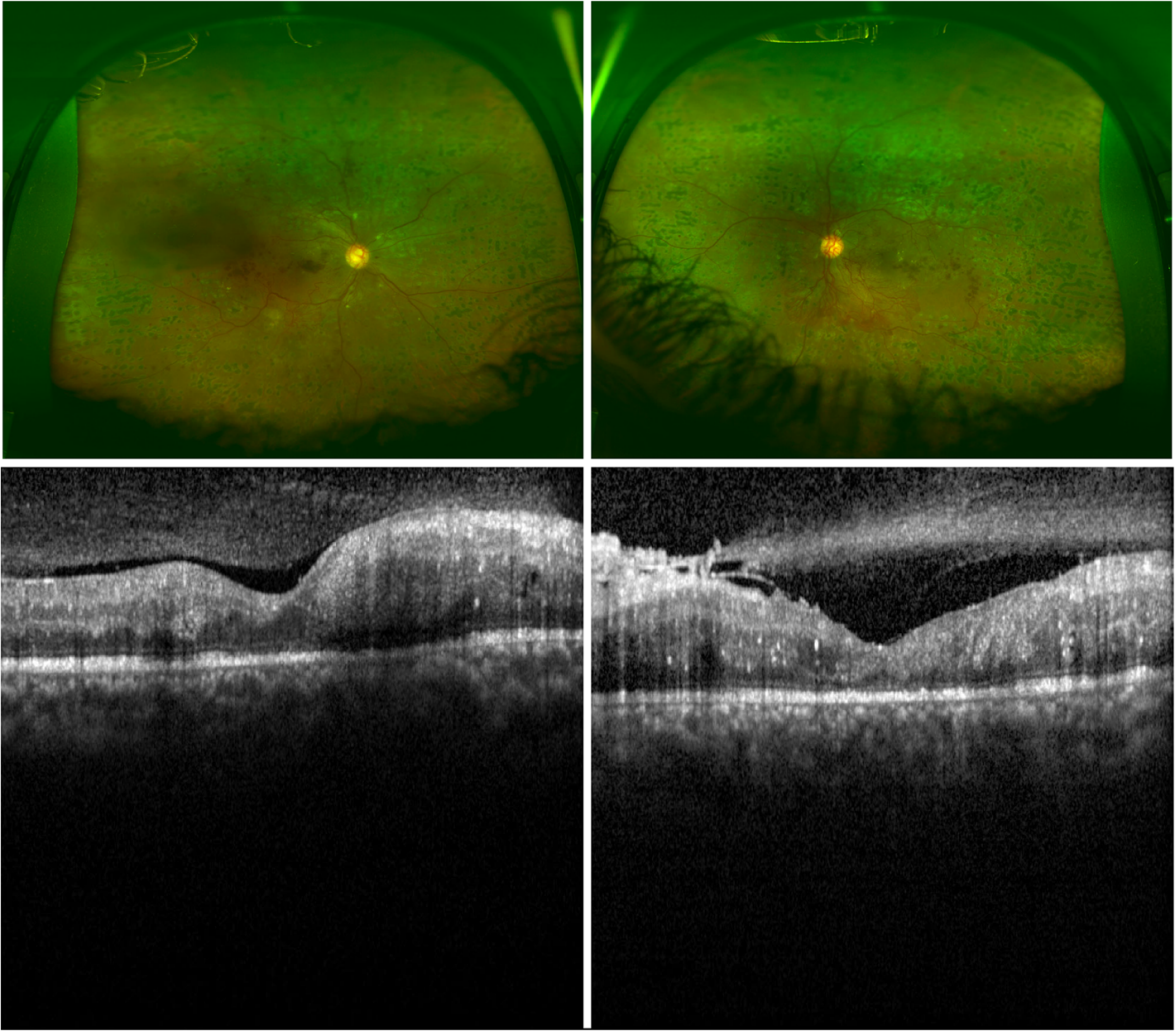
A 24-year-old man with a history of tobacco smoking and poorly regulated type 1 diabetes mellitus. *Upper panel*. Wide-angle imaging of his right and left eyes showing non-regressed neovascularizations in spite of widespread bilateral scarring from previous panretinal photocoagulation. *Lower panel*. Optical coherence tomography transfoveal single line scans of his right and left eyes show that there is no clinically significant macular edema and early epiretinal fibrosis in his left eye. He was offered intravitreal bevacizumab injections; however, he refused, and was instead given additional panretinal photocoagulation

Due to the unfavorable risk factor profile, he received several rounds of PRP in both eyes during the following 1 year after presentation; however, he developed PDR and the neovascularizations did not regress, in spite of the PRP (Fig. 1 upper panel). His unaided visual acuity was 20/200 improving to 20/50 with pinhole in his right eye and 8/200 improving to 20/70 with pinhole in his left eye. There was a discussion with our patient whether to proceed with additional PRP or to inject off-label intravitreal bevacizumab. A potential risk with intravitreal bevacizumab is the progression of fibrosis, which may ultimately lead to tractional RD [2, 3]. There were early signs of fibrosis in his left eye on optical coherence tomography (Fig. 1 lower panel).

**Fig. 2:**
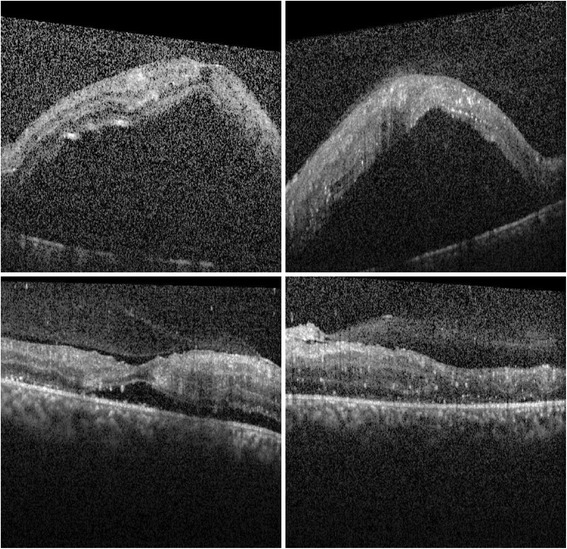
A 24-year-old man with a history of tobacco smoking and poorly regulated type 1 diabetes mellitus. *Upper panel*. Two days after the most recent bilateral augmentation of panretinal photocoagulation. Optical coherence tomography transfoveal single line scans of his right and left eyes show extensive serous retinal detachment. He was given bilateral 1.25 intravitreal bevacizumab injections. *Lower panel*. Eleven days after the intravitreal bevacizumab injections. Optical coherence tomography transfoveal single line scans of his right and left eyes show resolution of the subretinal fluid

He was recommended intravitreal bevacizumab injection, however he refused again. Instead, another round of PRP was attempted in both eyes. He presented 2 days later with bilateral extensive serous RDs involving the macula (Fig. 2 upper panel). His unaided visual acuity was 1/200 in his right eye and 5/200 in his left eye, with no improvement with pinhole correction. At this point, he accepted a single off-label injection of 1.25 mg bevacizumab (Avastin; Genentech, South San Francisco, CA, USA) in each eye. Eleven days after the bilateral injection, the serous RD had regressed completely in his left eye, and there was a substantial regression in his right eye (Fig. 2 lower panel), which subsequently regressed completely over the following 2 weeks. During the follow-up, his unaided vision gradually recovered up to 20/100 improving to 20/40 with pinhole in his right eye and 20/300 improving to 20/60 with pinhole in his left eye. At 3 months after the intravitreal bevacizumab injection, there was partial regression of the neovascularizations and no signs of tractional RD (not shown).

Imaging was performed using spectral domain optical coherence tomography (Heidelberg Engineering, Inc., Heidelberg, Germany) and wide-field fundus photography (Optos PLC, Dunfermline, UK).

PRP was performed using Pattern Scan Laser (PASCAL) photocoagulator (OptiMedia, Santa Clara, California, USA) and Volk QuadrAspheric fundus contact lens (Volk Optical Inc. Mentor, OH, USA). The duration was 20 ms for each spot with a spot size of 200 micrometers and spacing of 0.75 using a 2 × 2 or 3 × 3 grid pattern. The power range was 200 to 1000 milliWatt, adjusted as needed until retinal whitening was seen for each burn.

## Discussion

This case report highlights the clinical course seen during the progression of PDR in spite of several sessions of PRP. After one of these sessions, a bilateral serous RD appeared. This complication was successfully managed with intravitreal bevacizumab. To the best of our knowledge, the use of intravitreal bevacizumab has not been described previously for this complication. Serous RD after PRP for PDR is a rare complication nowadays and we are aware of only two publications from this century using high resolution imaging to describe its features. None of these, however, presented wide-angle imaging of the PDR characteristics. Gharbiya *et al*. [4] used bilateral intravitreal triamcinolone injections, leading to rapid resolution of the serous RD in a 52-year-old woman with type 2 DM. Azar *et al*. [5] used two bilateral subconjunctival betamethasone injections, leading to resolution of the subretinal fluid (SRF) and recovery of vision in a poorly regulated 34-year-old woman who had a miscarriage in the 20th week of pregnancy.

There are no studies on the natural history of the resolution of SRF after PRP using optical coherence tomography. Doft and Blankenship (1982) described 13 out of 50 eyes that developed serous RD after PRP, with resolution of the serous RD within 14 days in all eyes; however, this was not documented with optical coherence tomography [6].

The case described by Gharbiya *et al*. [4] most likely had persisting SRF for 3 weeks after PRP, with almost complete resolution within 1 week after the intravitreal triamcinolone acetonide (TA). In the case described by Azar *et al*. [5], SRF presented 2 days after the PRP, with resolution of SRF within 1 month after subconjunctival betamethasone injection. Thus we argue that the fast resolution of SRF described in this case may have been due to the intravitreal Avastin (bevacizumab) injection. This is supported by the fact that there was a partial regression of the neovascularizations and no signs of tractional RD at 3 months after the intravitreal bevacizumab (not shown). However, an element of spontaneous resolution of SRF cannot be excluded. The limitation of this study is that it is a single case report and there is no control group. However, serous RD is nowadays a very rare complication after PRP, and therefore its optimal management is unlikely to be supported by any clinical trials on DR.

## Conclusions

Serous RD after PRP for PDR is a rare complication nowadays. In this case, it seems that excessive photocoagulation exceeded the energy-absorbing capacity of the retinal pigment epithelium, leading to a disruption of the blood–retinal barrier. A single injection of bilateral intravitreal bevacizumab was sufficient to control the serous RD. This effect may have been due to a reduction of vascular leakage resulting from the mechanism of action of this drug. No complications were noted from the injection. To conclude, caution should be exerted when attempting bilateral PRP. This has to be weighed against issues such as the risk of progression of PDR with complications such as vitreous hemorrhage and patient compliance and access to ophthalmic health care.

## Abbreviations

BP: Blood pressure
DM: Diabetes mellitus
DR: Diabetic retinopathy
PDR: Proliferative diabetic retinopathy
PRP: Panretinal photocoagulation
RD: Retinal detachment
SRF: Subretinal fluid
